# Social media influence in the COVID-19 Pandemic

**DOI:** 10.1590/S1677-5538.IBJU.2020.S121

**Published:** 2020-07-27

**Authors:** Daniel A. González-Padilla, Leonardo Tortolero-Blanco

**Affiliations:** 1 University Hospital 12 de Octubre Department of Urology Madrid Spain Department of Urology, University Hospital 12 de Octubre, Madrid, Spain; 2 Hospital Universitario La Moraleja Madrid Spain Urology service, Hospital Universitario La Moraleja, Madrid, Spain

**Keywords:** Social Media, COVID-19 diagnostic testing [Supplementary Concept], Information Dissemination

## Abstract

Never before in human history has it been possible to communicate so quickly during a pandemic, social media platforms have been a key piece for the dissemination of information; however, there are multiple advantages and disadvantages that must be considered. Responsible use of these tools can help quickly disseminate important new information, relevant new scientific findings, share diagnostic, treatment, and followup protocols, as well as compare different approaches globally, removing geographic boundaries for the first time in history.

In order to use these tools in a responsible and useful way, it is recommended to follow some basic guidelines when sharing information on social networks in the COVID-19 era. In this paper, we summarize the most relevant information on the influence, and advantages, and disadvantages of the use of social networks during the COVID-19 pandemic.

## INTRODUCTION

Social media platforms are amongst the most widely used sources of information in the World, the easy and inexpensive access to the internet and a large number of registered users in these platforms make them one of the easiest and most effective ways to disseminate information. During major events, the overall response is usually a greater search for information be it a sports event, a disease, or a natural disaster.

A good example can be seen with the peak of searches for information on the Internet and social media platforms in China preceding the peak of incidence in COVID-19 cases by 10-14 days, with which Internet and social media networks searches have a demonstrated correlation with the incidence of disease (1, 2).

Social media platforms have also become helpful for the lay public to maintain communication with friends and family to reduce isolation and boredom which have been associated with anxiety and long-term distress, therefore becoming an important recommendation for isolation at home to help to reduce the psychological impact (3).

Some of the most relevant characteristics of social media platforms in this pandemic has been the rapid dissemination of protocols at regional, national, and international levels. Sharing protocols about treatment, personal protection equipment, or even proposals for fair allocation in scarce medical resource settings have now become the new normal (4).

This allows centers with less capacity to develop protocols at sufficient speed to be able to implement or adapt other's protocols to their particular situation or resources in minimal time, something unthinkable 20 years ago when most social media platforms had not yet been born (5). We provided in this manuscript, the most important advantages and disadvantages associated with the use of social media platforms during the pandemic.

### Advantages of social media use

Social media have the great advantage of rapid dissemination of educational content in the COVID-19 era, for example, Chan et al. (6) developed an infographic about airway management of patients with suspected or confirmed COVID-19. It was shared through Twitter and WeChat, in a few days requests were received for its translation into more than ten languages, besides the distribution allowed adapting the infographic to the particularities of each healthcare setting.

Faster dissemination of information regarding preventive measures has a lot of potentials. A recent study by Basch et al. (7) evaluated the 100 most viewed videos on YouTube with the word “coronavirus”, these together had more than 165 million views as of March 5, 2020, 85% of them belonging to news channels; It was found that less than ⅓ of the videos mentioned the recommended prevention measures, less than half mentioned the most frequent symptoms, however, almost 90% commented on deaths, anxiety, and the quarantine status. This study leaves us with an important reflection on the missed opportunities for dissemination of quality information on the prevention of contagion and frequent symptoms of COVID-19 on platforms such as YouTube, which are being increasingly consulted as an information source.

When it comes to publications, studies have shown that the dissemination of scientific literature on social media platforms (Facebook, Twitter, etc.) increases the number of downloads, queries, and citations of these articles (8–10) which, with the COVID-19 pandemic are characteristics that have undoubtedly allowed rapid dissemination of knowledge worldwide, in addition to markedly reduced editorial times, which have gone from months of processing to days or weeks since its reception.

For this reason, before sharing medical information, we advise following some guidelines of responsible use of social media when disseminating information; these guidelines are summarized in Table-1.

**Table 1:** Criteria for the responsible use of the information disseminated on social media. Modified from Chan et al. (6).

Guidelines for responsible use of social media for disseminating information
1 - Prefer dissemination through established professional platforms, or communication groups.
2 - Provide source when sharing information. Abstain from sharing information without a clear and trusted source.
3 - Abstain from sharing information that may only induce panic or anxiety.
4 - Quality should be preferred over quantity when sharing information, *In vitro* studies and low-quality evidence are of little or no use in daily practice and may give unfounded hope.
5 - Declare conflicts of interest, when appropriate.
6 - Avoid providing medical advice in social media and abstain from giving recommendations not backed by evidence as this may confuse lay public.
7 - Use transparent methods for peer review and feedback, like platforms for post-publication peer review processes or pre-print (unpublished manuscripts) like medRxiv.org, providing author/institutional contact, and pursue a traditional peer review process as soon as feasible.

Another advantage of social media platforms during the COVID-19 pandemic has been the possibility of arranging collaborative research projects, surveys, and multi-center studies. Finally, another advantage of social media platforms is supporting continued medical education through online live and recorded webinars through platforms like YouTube, Skype, or Zoom.

### Disadvantages of social media use

Among the disadvantages, we have the possibility that information transmitted is not current, has not been subjected to peer review, is invalid, incorrect, not applicable to our environment, or even false.

Another big obstacle for social media and the dissemination of information are the *“bubble filters”,* a concept coined by Eli Pariser in 2011 (11), which tells us about a “personalized ecosystem” towards the user, in which the algorithms through the data collected from the same user, predict their preferences and yield results that are considered similar to the likes of that user. These bubbles produce a loop of similar content that prevents the user from seeing other different sources to contrast information (12). This concept applies to any scenario or illness that is consulted in internet search engines or on social media platforms such as Facebook and Twitter.

Finally, probably the worst face of social media is the potential to disseminate erroneous, alarmist, and exaggerated information that can cause fear, stress, depression, and anxiety in people with or without underlying psychiatric illnesses.

A study by Wang et al. (13) in China, conducting an online survey with 1,210 responses, found that 53.8% of respondents considered the epidemic's psychological impact as moderate or severe; even a research group created and validated a scale called “Fear of COVID-19 scale” (14) to assess the level of stress and anxiety in the population and to establish appropriate measures to prevent sequels associated, such as post-traumatic stress disorder (PTSD) which was the most prevalent psychiatric sequelae after the Severe Acute Respiratory Syndrome (SARS) epidemic in Asia in 2003, followed by depressive disorders (15). Other more severe diseases or events such as suicides have already been reported in some parts of the World like India, Britain, Germany, and Italy (16).

### Infodemic and disinformation

By April 30, 2020, there were more than 8,000 papers in PubMed with the word “COVID-19” (17), which tells us about the tsunami of information in less than 4 months since its appearance in China; with all the attention poured into the media, the avalanche of data becomes unaffordable, something also called “Infodemia” (18, 19).

On the other hand, at the same speed information travels, disinformation does, it is for this same reason that some authors have suggested creating working groups aimed at fighting myths and disinformation in social media platforms (20). In this way, World Health Organization (WHO) developed an exclusive section on its website designed for coronavirus myth-busting (21).

Connected with this same issue, the lay public gains access to preliminary and *in vitro* study results through newscasts practically at the same time that this information is available to the medical community, which combined with the generalized fear of the virus and healthcare systems overwhelmed, generates pressure on patients to demand such experimental treatments for themselves or their families, and doctors may feel compelled to try them, even when there is no high-quality evidence to support their use for these purposes.

## CONCLUSIONS

Social media has advantages and disadvantages, the responsible use of these tools can help during a pandemic to quickly spread new important information, sharing diagnostic, treatment and follow-up protocols, comparing different approaches from other parts of the World to adapt them to our setting and available resources, with the downside of possible dissemination of fake data, myths, and pessimist information that combined with quarantine states may lead to anxiety, depression and in some extreme cases, the suicide. Therefore, it is advisable not to contribute to the infodemic and follow a responsible use of social media when disseminating information.

## ABBREVIATIONS

PTSD: post-traumatic stress disorder
SARS: Severe Acute Respiratory Syndrome
WHO: World Health Organization
